# Myeloperoxidase enzyme-catalyzed breakdown of zero-dimension carbon quantum dots

**DOI:** 10.3389/fmedt.2024.1493288

**Published:** 2024-11-28

**Authors:** Pooja Singh, Lalit Kumar Singh

**Affiliations:** Department of Biochemical Engineering, School of Chemical Engineering, Harcourt Butler Technical University, Kanpur, India

**Keywords:** myeloperoxidase enzyme, photoluminescence, hypochlorous acid, biodegradation, peroxidase, neutrophil granulocytes, carbon quantum dots

## Abstract

Carbon quantum dots (CQDs) have shown considerable interest in multiple fields including bioimaging, biosensing, photocatalysis, ion sensing, heavy metal detection, and therapy due to highly tunable photoluminescence and good photostability. Apart from having optical properties CQDs offer several advantages such as low toxicity, environmental friendliness, affordability, and simple synthesis methods. Furthermore, by modifying their surface and functionality, it's possible to precisely control their physical and chemical characteristics. Nevertheless, the growing utilization of carbon-based nanomaterials (CNMs) requires thorough examination of their potential toxicity and long-term impacts on human health and biological systems. In this study, carbon quantum dots (CQDs) were synthesized via a microwave-assisted method using citric acid and urea as precursors, resulting in an average particle diameter of 10.73 nm. The CQDs were further characterized using SEM and FTIR analysis. The CQDs exhibited an excitation wavelength of 320 nm, displaying an emission peak at 430 nm. The enzymatic biodegradation of CQDs by human myeloperoxidase enzyme has been thoroughly investigated here. It is very crucial to understand how these carbon quantum dots interact with the innate immune system that plays a vital role in recognizing and clearing foreign particles. Human myeloperoxidase (MPO), a key enzyme highly expressed in neutrophil granulocytes during inflammatory responses, has been shown to facilitate the biodegradation of carbon quantum dots and various carbon-based nanomaterials through oxidative processes. As a member of the peroxidase family, MPO produces hypochlorous acid (HOCl) and a range of reactive intermediates to eliminate pathogens. Consequently, the study of the biodegradability of CQDs within biological systems is essential for accelerating technological advancements. Here, we have assessed breakdown of CQDs through an oxidative process facilitated by a myeloperoxidase (MPO)-based peroxide system. The human MPO enzyme acted as a catalyst for the CQD degradation, and the addition of hydrogen peroxide (H_2_O_2_) and sodium chloride (NaCl) was found to accelerate the reaction.

## 1 Introduction

Carbon-derived nanomaterials, including carbon quantum dots (CQDs), fullerenes, graphene quantum dots, and carbon nanotubes, have garnered significant attention in recent times for a variety of applications within the fields of science and technology, attributable to their exceptional optical, mechanical, and photoluminescent characteristics (1, 2). With the pervasive utilization of carbon-derived nanomaterials, their inevitable release into the environment raises concerns regarding potential environmental contamination (3, 4). Consequently, the toxicity associated with nanomaterials has garnered increased scrutiny in recent years. When metallic carbon nanomaterials accumulate within cellular structures, they can produce free radicals that lead to DNA damage, inflammation, and oxidative stress (5). Thus, the potential hazards posed by engineered nanomaterials, including graphene quantum dots, carbon quantum dots, and metal oxide nanoparticles, remain a significant concern. In response to these issues, recent advancements have focused on developing engineered nanomaterials characterized by low toxicity and minimal environmental impact. Notably, carbon quantum dots (CQDs), representing a novel class of carbon-based engineered nanomaterials, have garnered substantial interest due to their ease of synthesis and modification, high biocompatibility, and versatility in application (4, 6).

Carbon nanoparticles (CQDs) are extremely small nanoparticles made of carbon that have undergone surface passivation or other modifications to become functionalized. The structure of CQDs can be both amorphous as well as crystalline (7). They exhibit sp^2^ carbon hybridization, however sp^3^ hybridization has occasionally also been observed (2, 8, 9).

CQDs are zero-dimensional carbon nanomaterials that are limited in size both in-plane and out-of-plane and distinguished by their relatively strong fluorescence properties and small size (less than 10 nm) (10). The fluorescence characters in CQDs are due to two types of sources one is fluorescence emission from the bandgap transitions within conjugated domains and fluorescence arising from surface defects. The CQDs are well-suited for biomedical applications due to their fluorescence emission in the near-infrared region (11). By changing the excitation wavelength, these structures allow for the tuning of the fluorescence. As the use of carbon quantum dots increases, it is crucial to closely examine their effects on human health and biological systems (12, 13). It is especially crucial to comprehend how they interact with innate immune system cells (12, 14, 15).

Carbon nanomaterials undergo biodegradation both *in vivo* and *in vitro* via myeloperoxidase (MPO), an essential enzyme that is secreted by neutrophils during inflammation (8, 16, 17). The capability of peroxidase enzymes to degrade carbon nanomaterials has emphasized the significance of using an ecologically safe enzymatic carbon nanomaterial degrading approach (18, 19). The human myeloperoxidase enzyme is a heme-containing complex that is well-known for having two glycosylated and two unglycosylated chains. This myeloperoxidase enzyme generates hypochlorous acid (HClO) molecules from hydrogen peroxide (H_2_O_2_) and chloride (Cl^−^) (20, 21). When carboxylated carbon nanomaterials are incubated with MPO, H_2_O_2_, and Cl^–^ a variety of carbonaceous byproducts, such as CO, CO_2_, and various hydrocarbons are produced (8, 22). The structure and enzymatic characteristics of MPO enzyme is because of distinct prosthetic groups (23). When the enzyme MPO-Fe (III) is secreted, it undergoes a fast and reversible reaction with H_2_O_2_ to generate Compound I, an intermediate with a Fe group that has undergone two-electron oxidation. In the presence of H_2_O_2_, this redox intermediate transform halide (Cl^−^, Br^−^) and pseudohalides (SCN^−^) to their corresponding hypohalous acids (20, 24). This biochemical process, referred to as the halogenation cycle, generates Cl^−^ ions as the preferred substrate and hypochlorous acid (HOCl), which serves as the dominant oxidant under physiological circumstances (23, 25).

However, carbon quantum dots (CQDs) are predominantly regarded as highly biocompatible and safe engineered nanomaterials (ENMs), it remains crucial to thoroughly investigate their potential toxicity before widespread use and mass production. Most of the research has conducted experiments at the cellular level to assess various types of CQDs, demonstrating that these structures exhibit lower cytotoxicity and enhanced biocompatibility compared to metallic ENMs (3, 5). Some investigations have employed animal models to assess the toxicity associated with CQDs (26). A considerable number of existing quantum dots are composed of hazardous metals such as cadmium and lead, which constrains their applicability in electronic and medical devices. This research focused on synthesizing carbon quantum dots through a single-step process, utilizing urea and citric acid as substrates and employing microwave-based synthesis method. Here, we have investigated various properties of the synthesized carbon quantum dots, including their size distribution, zeta potential, and chemical composition employing ultraviolet (UV) spectroscopy and dynamic light scattering (DLS) techniques. Additionally, we have examined the PL and UV absorption properties of CQDs using fluorescence and UV scans.

## 2 Materials and methods

### 2.1 Reagents

The citric acid and urea were obtained from HiMedia Laboratories Pvt. Ltd., India. The DMF (N, N-Dimethylformamide) was purchased from Merck Millipore. Hydrogen peroxide was obtained from SRL Pvt. Ltd., India. A Milli-Q device (Millipore, Burlington, MA, USA) provided the deionized water. Human origin myeloperoxidase (MPO) was purchased from Merck, Rahway, NJ, USA.

### 2.2 Preparation of urea and citric acid derived carbon quantum dots

The functionality and photoluminescence characteristics of carbon quantum dots can be precisely controlled by adjusting the surface functional groups and chemical configurations (27). A comprehensive review by (7) have explored into the synthesis of CQDs highlighting the potential for functionalization in accordance with their application requirements. Carbon quantum dots synthesis may be achieved through two distinct strategies: one is a top-down approach, where larger carbon-based materials are systematically reduced in size to form quantum dots. The second approach, called bottom-up, takes the opposite route by assembling quantum dots from smaller carbon-containing molecules or compounds (28, 29).

**Figure 1:**
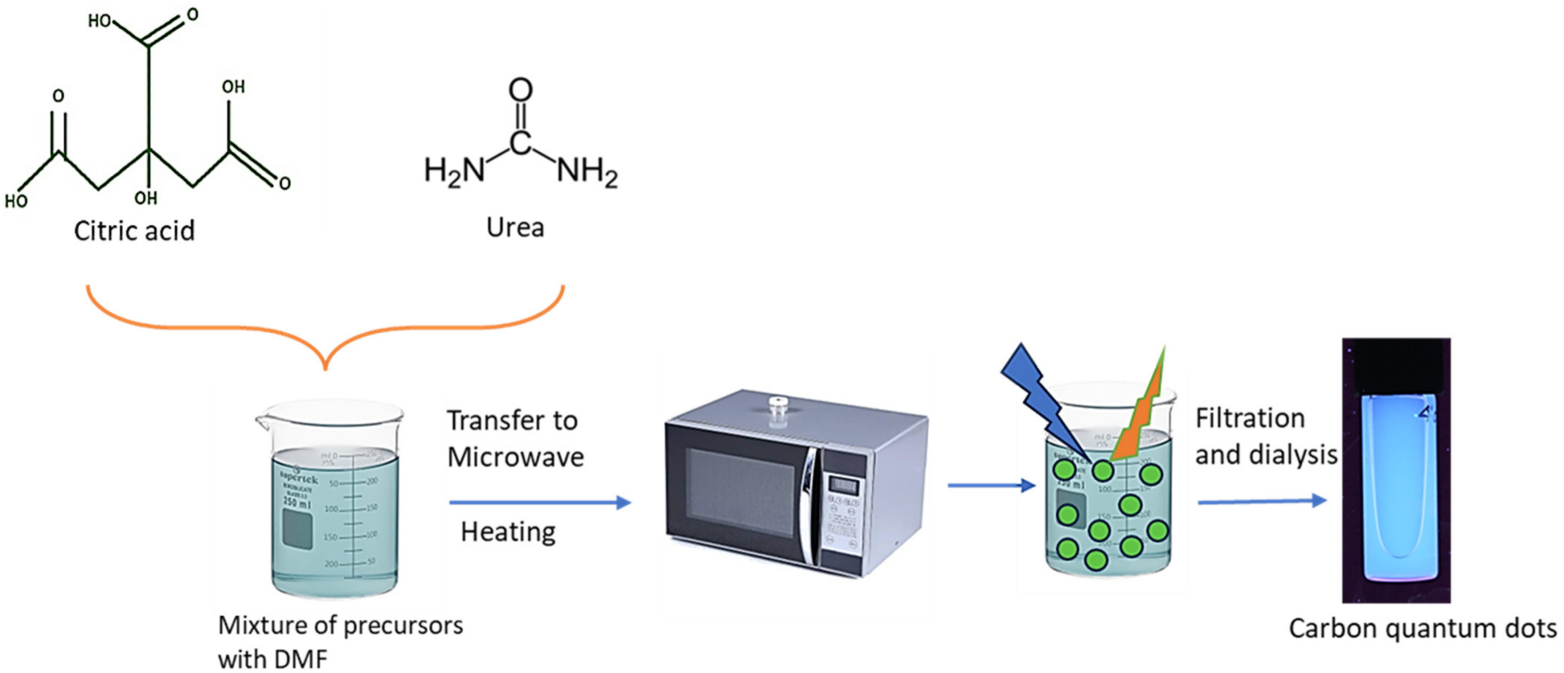
Synthesis of carbon quantum dots from urea and citric acid.

Here, the microwave-assisted synthesis method was utilized to prepare CQDs using urea and citric acid as the starting materials. Equal amounts of citric acid and urea, 5.0 grams each, were combined to create a 1:1 mixture for the synthesis of CQDs (30). Then this mixture was supplemented with 2 ml of N, N-dimethylformamide (DMF) solution. The prepared mixture was then microwaved at full power for two minutes using a standard domestic microwave. This process resulted in a charred substance with a chestnut brown color. After allowing this mixture to cool to room temperature, it was diluted with 50 ml of highly purified (Milli Q) water (31). The mixture was thoroughly blended using a high-speed homogenizer at 12,000 rpm for 15 min. The homogenized solution was centrifuged at 10,000 rpm for 40 min to remove larger particles, lumps, and residues from the CQDs. Following centrifugation, the pellet was discarded, and the supernatant was collected for subsequent processing (Figure 1). The supernatant was then dialyzed using 1 KDa dialyzer overnight at room temperature under continuous stirring. The dialyzed CQDs were then dried using a lyophilizer and were kept for long-term storage at 4°C.

### 2.3 Characterization methods for carbon quantum dots

For the physicochemical characterization of CQDs, 100 *µ*g/ml of CQDs were suspended in Milli Q water to disperse the CQDs. The suspension was sonicated thoroughly in a bath sonicator (Ikon Industries) for 15 min with ice-cold water. Malvern Zetasizer Advance Series (Malvern, UK) instrument was used for the analysis of the particle size distribution and zeta potential at room temperature to maintain the stability of CQDs. The resulting graph and data were analyzed using zetasizer associated software.

### 2.4 Fluorescence spectroscopy

UV absorption scans (200–400 nm) and fluorescence spectrum scans (300–750 nm) of carbon quantum dots were conducted using an Envision device (PerkinElmer, Waltham, MO, USA). The resulting spectra were then analyzed with SpectraGryph software.

### 2.5 Fourier-transform infrared spectroscopy (FT-IR)

During this research, The KBr disk approach was specially used to generate Fourier-transform infrared spectra utilizing a Mattson 5,000 spectrometer (Unicam, United Kingdom).

### 2.6 Scanning electron microscopy

For surface topology, scanning electron microscope is used through the utilization of an accelerating voltage ranging from 10–15 kV.

### 2.7 Biodegradability of synthesized carbon quantum dots

When carrying out the enzymatic biodegradation of CQDs, we implemented the methodology outlined by (32). An aliquot of 1 ml of Milli-Q water (autoclaved), containing a concentration of 140 mM NaCl, was employed in the suspension of 5 *μ*g/ml of human-derived MPO, which was subsequently combined with 50 *μ*g of CQDs (resulting in a final concentration of 100 *μ*g/ml). Subsequently, H_2_O_2_ was then added to the mixture at a rate of 100 *μ*M h^−1^ for 24 h (32). Following an interval of six hours, the MPO enzyme was reintroduced, and the reaction mixture was sustained at a controlled temperature of 37°C (33). Subsequently, equivalent volumes of CQDs were dispersed in 1 ml of autoclaved Milli-Q H_2_O and subjected to a treatment duration of 24 h, with or without the addition of H_2_O_2_, at a rate of 100 M h^−1^. Upon the completion of reaction period, the samples were subjected to an analytical procedure to evaluate their absorbance within the wavelength range of 300–500 nm utilizing a UV-1800 spectrometer (Shimadzu, Kyoto, Japan). The final spectra were then processed using SpectraGryph software.

## 3 Result and discussion

### 3.1 Preparation and physicochemical characterization methods of carbon quantum dots

**Figure 2:**
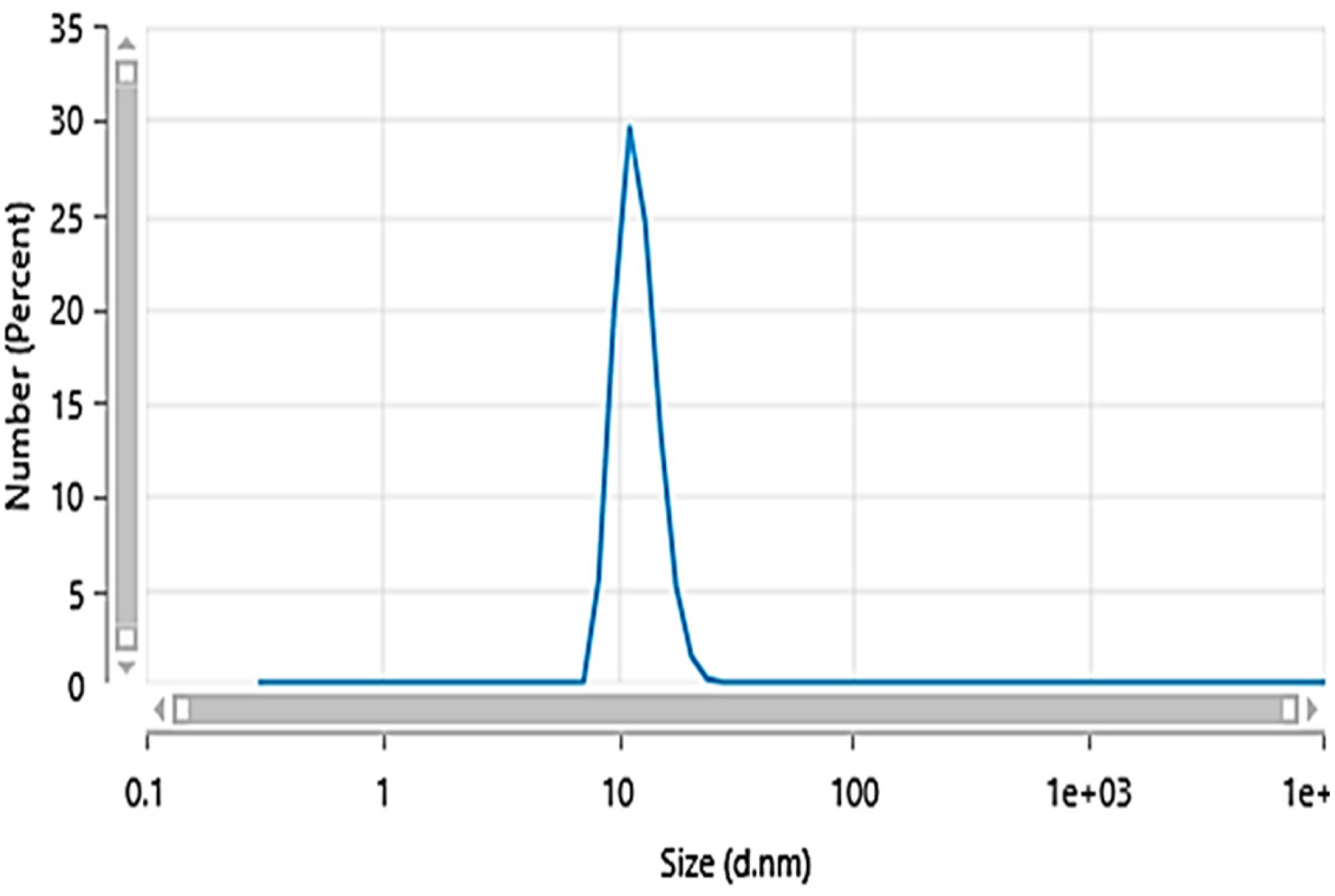
Particle size distribution.

**Figure 3:**
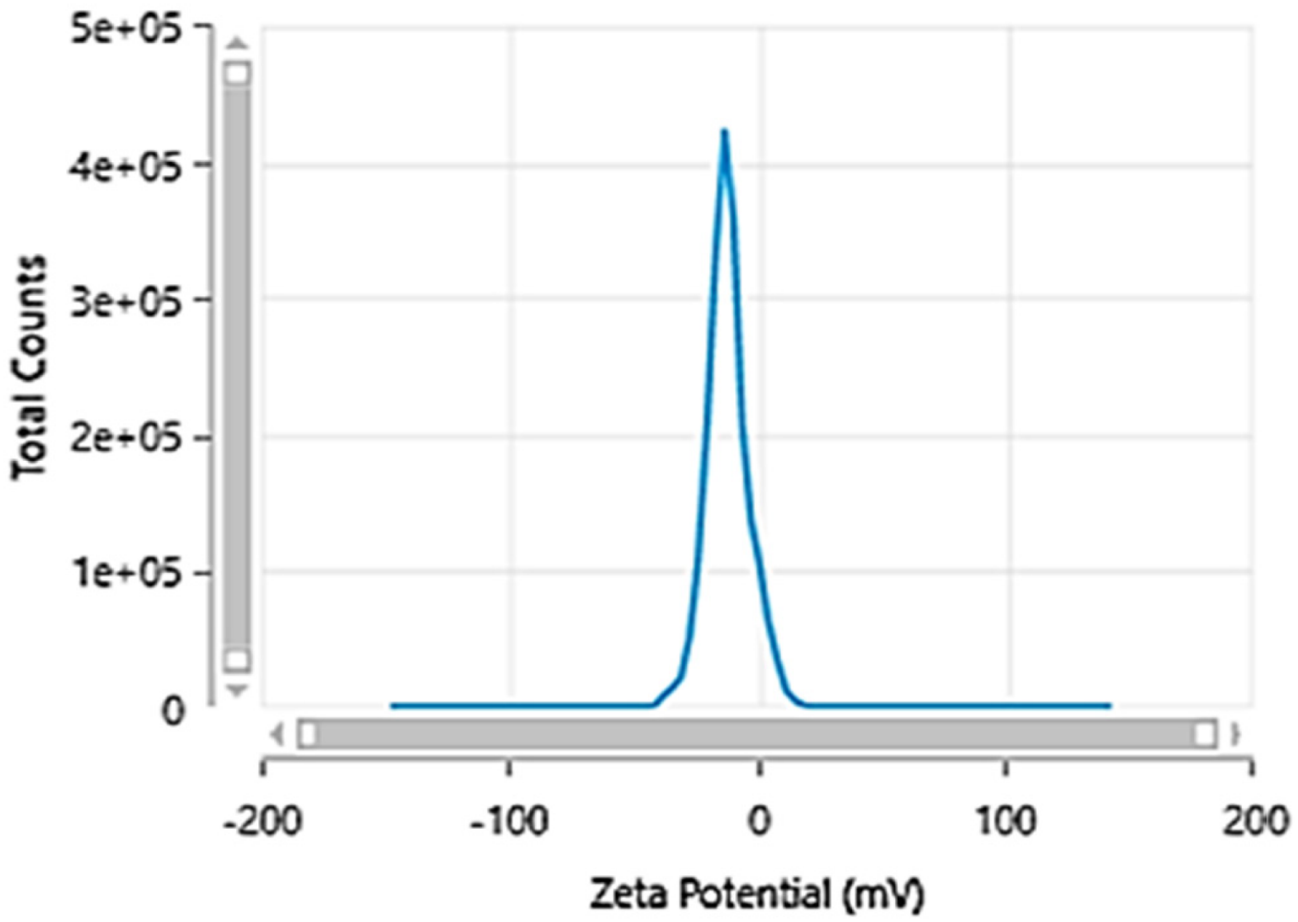
Zeta potential graph of carbon quantum dots.

**Figure 4:**
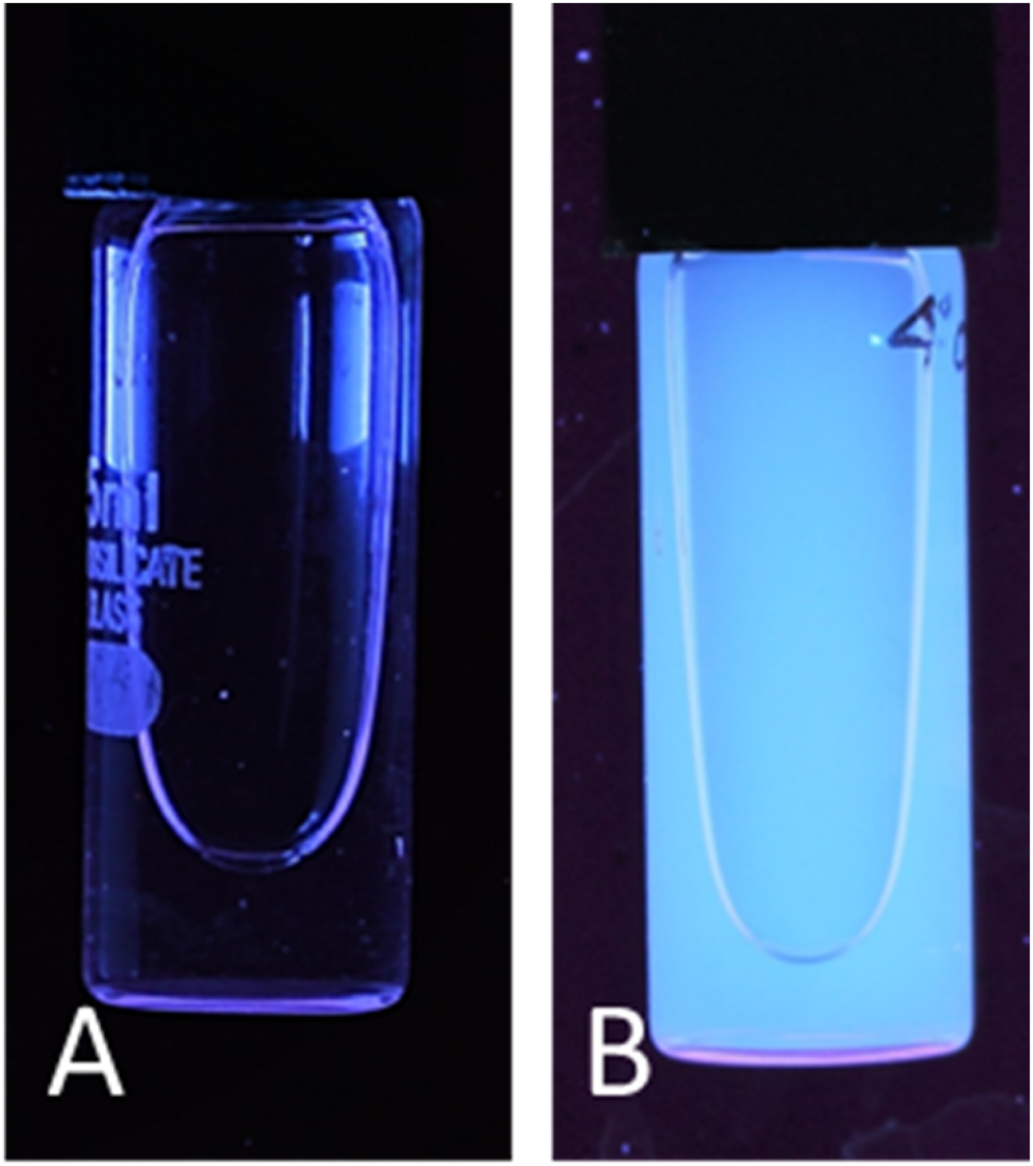
Carbon quantum dot photoluminescence in the presence of white light **(A)** and UV excitation **(B)**.

Carbon quantum dots were synthesized utilizing a 1:1 mixture of urea and citric acid through a simple single-step microwave-assisted approach using a 900-watt domestic microwave. The resulting CQDs were characterized using dynamic light scattering (DLS) to reveal a narrow size distribution with an average hydrodynamic diameter of 10.73 ± 0.18 nm (Figure 2), indicating the formation of uniform, nano-sized particles. The zeta potential was determined to be −11.9 ± 2.7 mV, suggesting colloidal stability of the CQDs in aqueous suspension (Figure 3). Furthermore, Carbon Quantum Dots (CQDs) were stimulated within the ultraviolet B (UVB) spectrum ranging from 280–320 nm. The resultant CQDs exhibited a photoluminescence (PL) emitting a blue color, as depicted in Figure 4 which is absent in standard white light sources. Neither chemical precursors nor dimethyl formamide shows any blue photoluminescence. This blue photoluminescence exhibited by CQDs arises from a combination of factors, including emissive traps, aromatic conjugate structures, triplet carbenes at zigzag edges, and surface functional groups such as carboxyl and oxygen-rich hydroxyl moieties (34).

### 3.2 Fluorescence spectroscopy analysis

**Figure 5:**
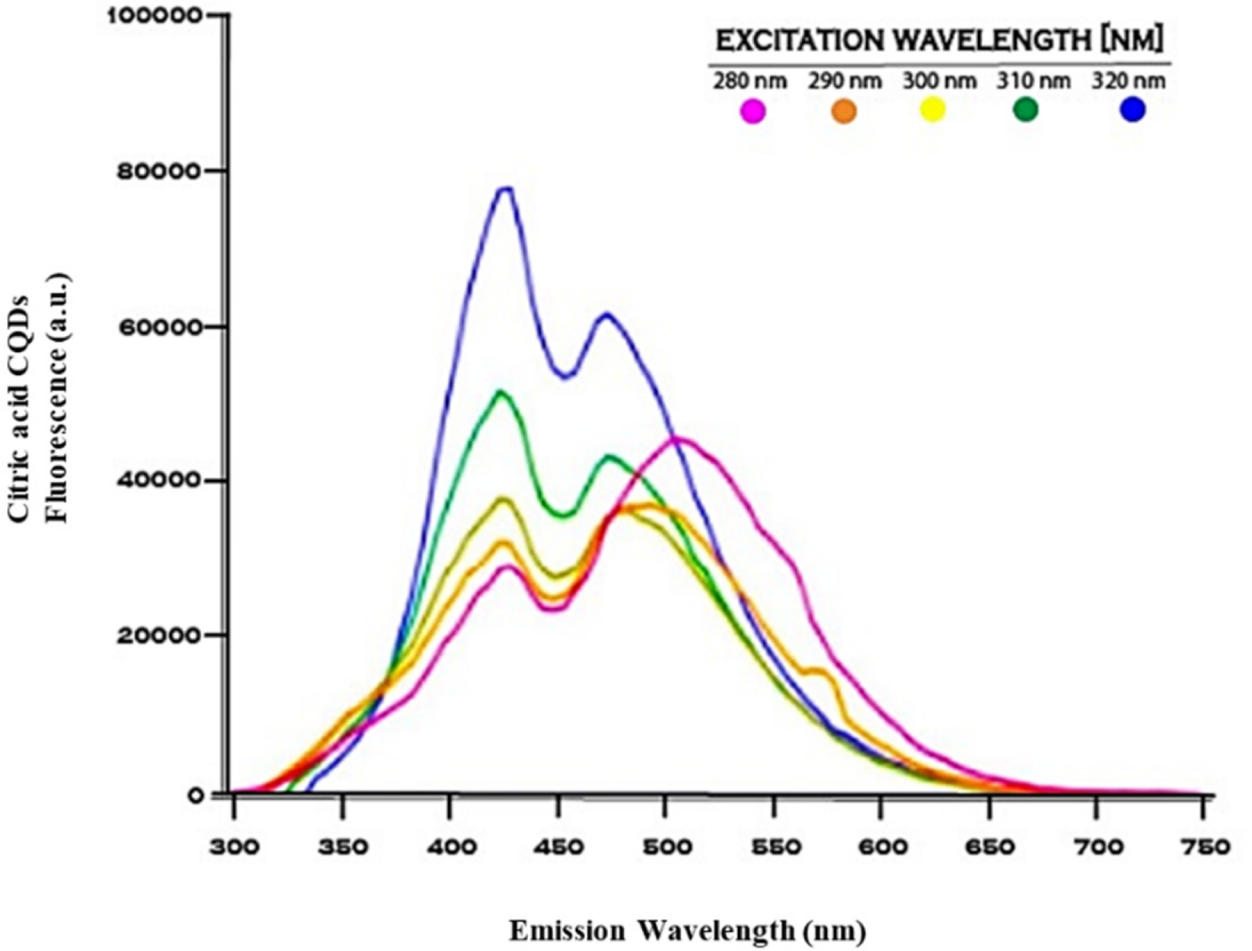
Fluorescence excitation and emission spectra of citric acid derived CQDs.

The maximum photoluminescence emission observed from the citric acid derived carbon quantum dots was identified at an excitation wavelength of 320 nm, displaying an emission peak at 430 nm (Figure 5). The photoluminescence characterized by blue color observed in carbon dots (CDs) is significantly modulated by the existence of emissive trapping sites, the presence of triplet carbenes situated at the zigzag boundaries, the configuration of aromatic conjugated frameworks, and the incorporation of functional moieties such as carboxyl groups and hydroxyl groups that are abundant in oxygen, all of which contribute to this intricate phenomenon (31).

### 3.3 Fourier-transform infrared spectroscopy (FT-IR) analysis

**Figure 6:**
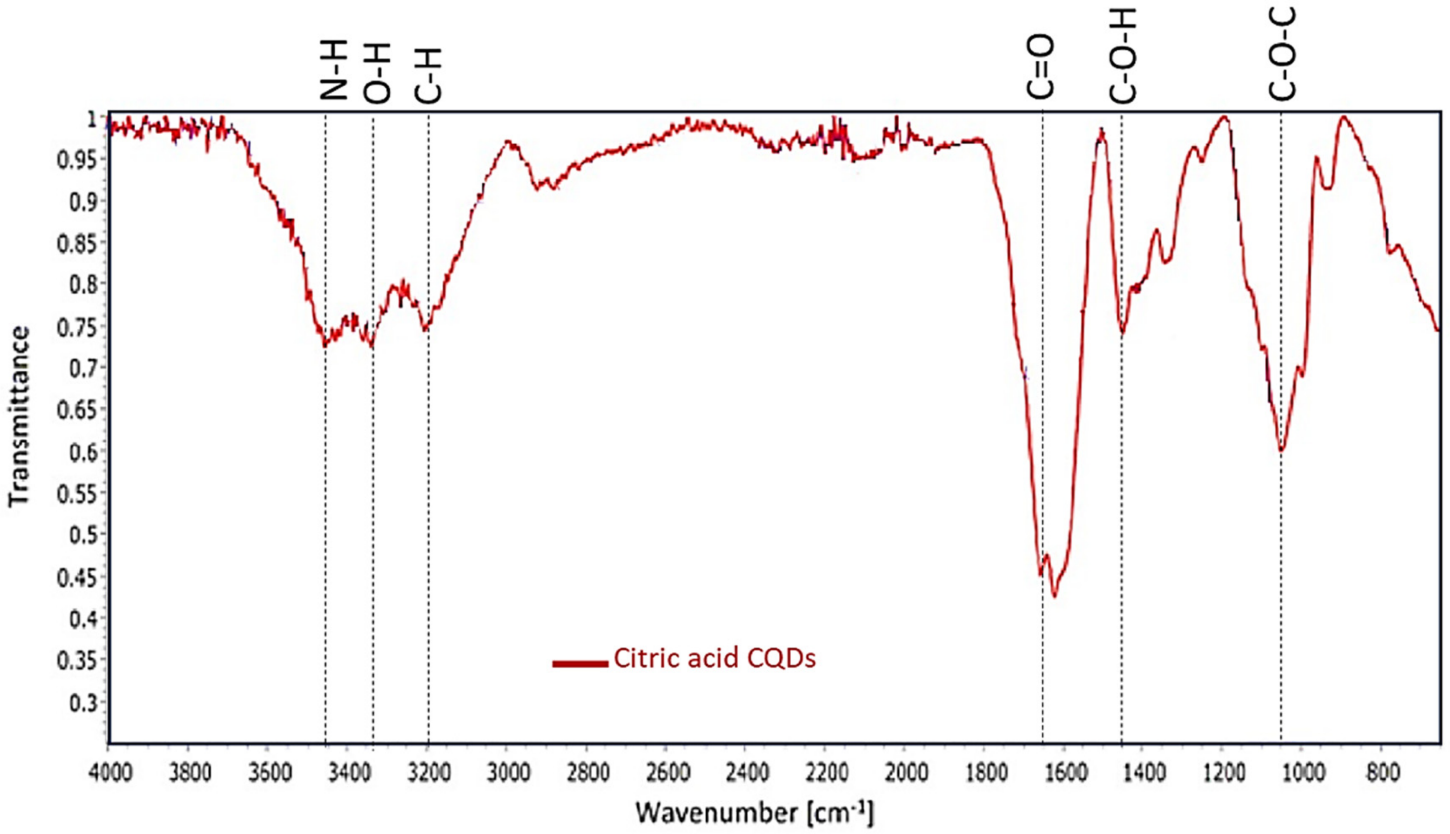
FTIR spectrum of citric acid derived CQDs.

The FTIR analysis conducted on the citric acid-derived carbon quantum dots (CQDs) elucidated the existence of N–H and O–H functional groups within the spectral range of 3,463–3,186 cm^−1^, along with a distinctive C=C bond, observed at 1,662 cm^−1^, while C–H stretching revealed at 2,920 cm^−1^. The spectrum also exhibited C–O–H vibration bands at 1,412 cm^−1^, C=O stretching at 1,600 cm^−1^, and C–O–C linkages at 1,069 cm^−1^ (Figure 6).

### 3.4 Scanning electron microscope analysis

**Figure 7:**
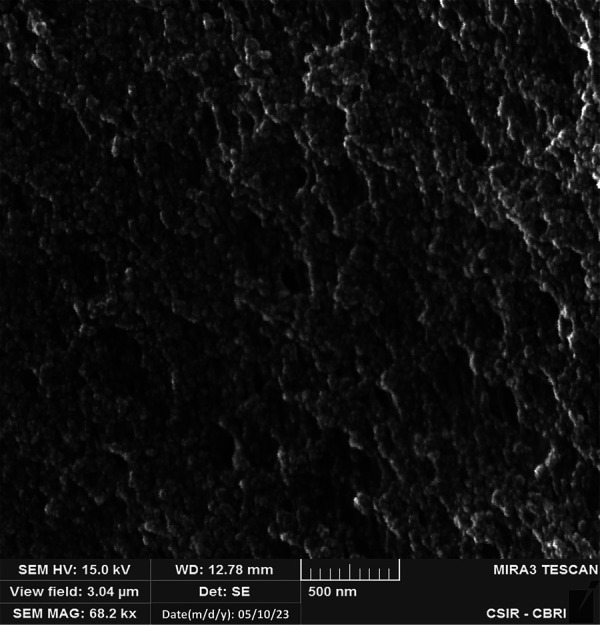
SEM images of citric acid derived CQDs.

SEM images illustrated the citric acid derived carbon quantum dots exhibited crumpled structures formed by precipitation, devoid of any visible dots due to their minute particle size and the tendency to agglomerate because of prolonged sample storage (Figure 7).

### 3.5 Enzymatic biodegradability of CQDs

**Figure 8:**
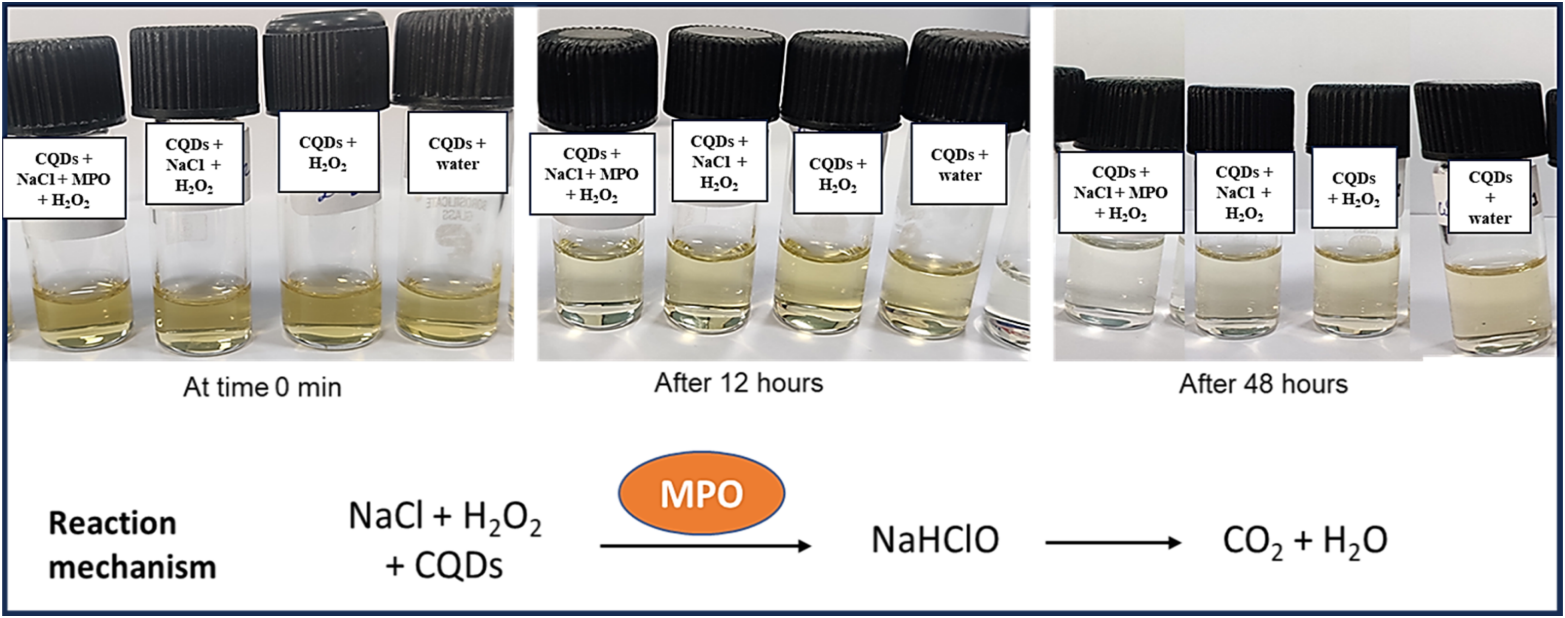
Incubation of CQDs with MPO, NaCl and H_2_O_2_ for different time intervals (0 min, 12 h, and 48 h).

**Figure 9:**
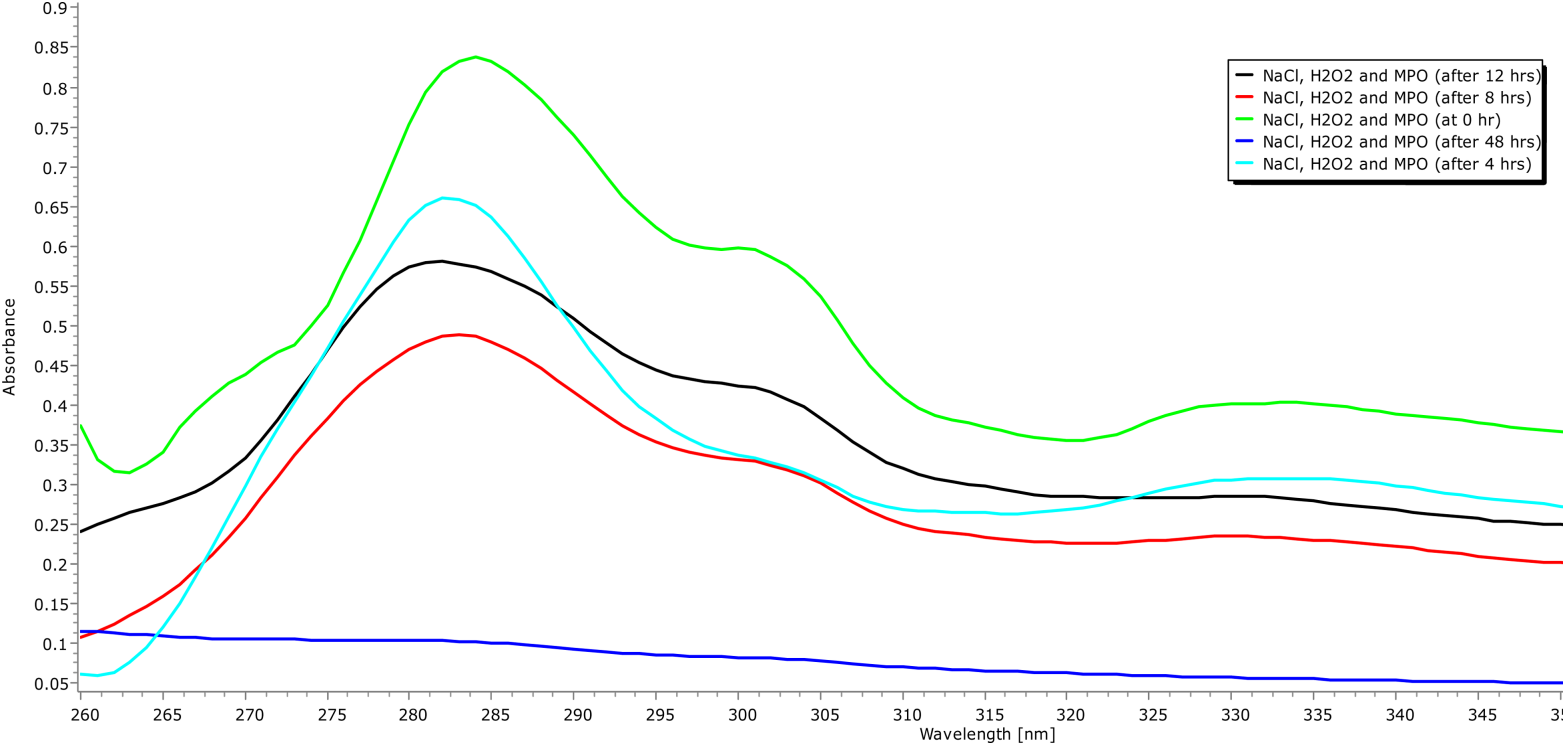
UVB absorption spectra (260–350 nm) of carbon quantum dots treated with H_2_O, H_2_O_2_, and human origin MPO, with NaCl, H_2_O_2_ for 24 h.

In addition to demonstrating their biocompatibility, it is necessary to investigate the biodegradability of carbon quantum dots. Human myeloperoxidase, along with various other peroxidases, possesses the enzymatic capability to decompose the graphitic matrix of carbon quantum dots (CQDs) and other carbon-based nanomaterials via an oxidative mechanism (35). Therefore, an MPO-based peroxide system was used to investigate the oxidative biodegradation of CQDs. When CQDs were incubated for 24 h with 140 mM NaCl, 5 *μ*g/ml MPO, and 100 *μ*M H_2_O_2_. In addition, we also performed control experiments where we incubated the CQDs exclusively with H_2_O_2_ to investigate any potential structural changes. Now the aliquots of the various samples were taken at 0, 4, 12, and 24 h intervals during incubation. Using UV-Vis spectroscopy, the absorbance of these aliquots was measured at various time intervals, and it was discovered that absorption decreased with time (Figures 8, 9).

The oxidative degradation of the CQDs caused by H_2_O_2_ alone with NaCl was comparatively less than that caused by the H_2_O_2_-driven peroxide system, MPO, and NaCl (Figure 8). To facilitate the synthesis of sodium hypochlorous acid (NaHOCl), which subsequently disintegrates the graphitic lattice, myeloperoxidase (MPO) interacts with the hydroxyl (OH) groups located on the surface of CQDs situated in proximity to defect sites. The OH group that present on the surface of the CQDs had come from citric acid verified by FTIR spectra (Figure 6). This observation is particularly relevant in the context of the body's innate immune defence mechanism. The primary barrier against invading pathogens and foreign materials consists of resident macrophages and neutrophils, which produce a myeloperoxidase-mediated peroxide system (22).

**Figure 10:**
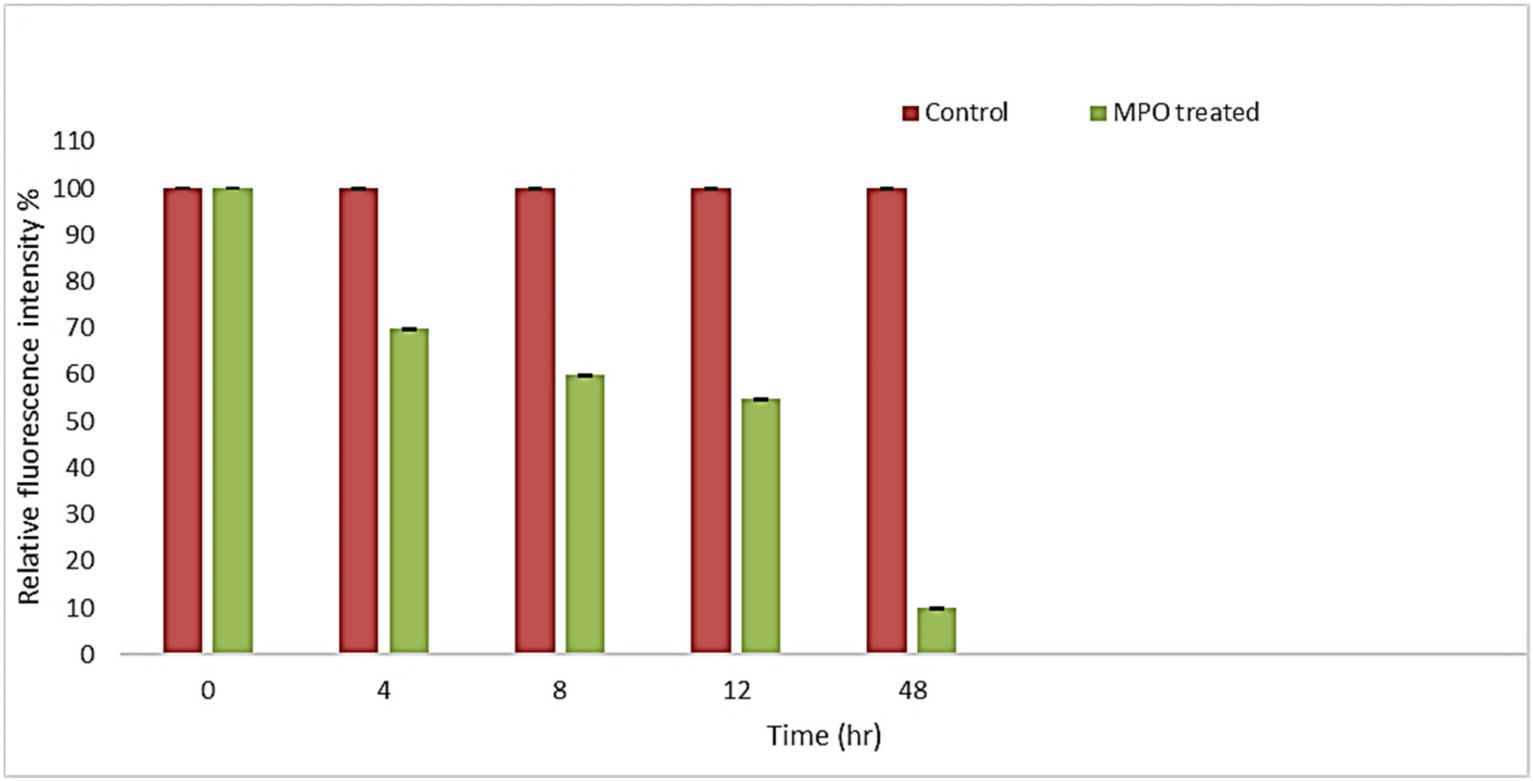
Fluorescence intensity of MPO treated and their respective control samples at different time intervals.

Additionally, the relative fluorescence intensity of CQDs was assessed over time, revealing a significant reduction in each MPO enzyme-treated sample's fluorescent intensity compared to its respective control (Figure 10). The most significant decrease in fluorescence intensity occurred during the first 4 h of incubation, in contrast to the subsequent time intervals. This phenomenon can likely be attributed to the high concentration of oxygenated functional groups present on the surface of the CQDs, which enhances their water dispersibility and facilitates interaction with the enzyme (35). We propose that after degrading the defective regions of the CQDs, the enzyme began to break down the more graphitic areas, potentially explaining the decreased rate of degradation after 4 h.

## 4 Conclusions

It was discovered that MPO based peroxide system significantly degraded the carbon quantum dots in the presence of H_2_O_2_ and MPO in the duration of the proposed experiment, (48 h). By using UV-vis spectroscopy, it was possible to confirm that the CQDs were biodegrading over time. At first, after 4 h of incubation, signs of degradation were seen, but after 48 h, they became more evident. Additionally, fluorescence intensity graph also confirmed that CQDs were degraded, and the fluorescence intensity decreased over time. Therefore, the results support the biocompatibility of CQDs for their use in various fields like biomedical and therapeutic applications.

## Data Availability

The original contributions presented in the study are included in the article/Supplementary Material, further inquiries can be directed to the corresponding author.
